# Computing infection distributions and longitudinal evolution patterns in lung CT images

**DOI:** 10.1186/s12880-021-00588-2

**Published:** 2021-03-23

**Authors:** Dongdong Gu, Liyun Chen, Fei Shan, Liming Xia, Jun Liu, Zhanhao Mo, Fuhua Yan, Bin Song, Yaozong Gao, Xiaohuan Cao, Yanbo Chen, Ying Shao, Miaofei Han, Bin Wang, Guocai Liu, Qian Wang, Feng Shi, Dinggang Shen, Zhong Xue

**Affiliations:** 1 Hunan University, Changsha, China; 2 Shanghai Jiao Tong University, Shanghai, China; 3 Shanghai United Imaging Intelligence Co., Ltd, Shanghai, China; 4 Shanghai Public Health Clinical Center, Shanghai, China; 5 Tongji Hospital, Wuhan, China; 6 Second Xiangya Hospital of Central South University, Changsha, China; 7 China-Japan Union Hospital of Jilin University, Changchun, China; 8 Ruijin Hospital, Shanghai, China; 9 West China Hospital of Sichuan University, Chengdu, Sichuan China

**Keywords:** COVID-19, Segmentation, Registration, Coronavirus infections, Lung, Probability

## Abstract

**Background:**

Spatial and temporal lung infection distributions of coronavirus disease 2019 (COVID-19) and their changes could reveal important patterns to better understand the disease and its time course. This paper presents a pipeline to analyze statistically these patterns by automatically segmenting the infection regions and registering them onto a common template.

**Methods:**

A VB-Net is designed to automatically segment infection regions in CT images. After training and validating the model, we segmented all the CT images in the study. The segmentation results are then warped onto a pre-defined template CT image using deformable registration based on lung fields. Then, the spatial distributions of infection regions and those during the course of the disease are calculated at the voxel level. Visualization and quantitative comparison can be performed between different groups. We compared the distribution maps between COVID-19 and community acquired pneumonia (CAP), between severe and critical COVID-19, and across the time course of the disease.

**Results:**

For the performance of infection segmentation, comparing the segmentation results with manually annotated ground-truth, the average Dice is 91.6% ± 10.0%, which is close to the inter-rater difference between two radiologists (the Dice is 96.1% ± 3.5%). The distribution map of infection regions shows that high probability regions are in the peripheral subpleural (up to 35.1% in probability). COVID-19 GGO lesions are more widely spread than consolidations, and the latter are located more peripherally. Onset images of severe COVID-19 (inpatients) show similar lesion distributions but with smaller areas of significant difference in the right lower lobe compared to critical COVID-19 (intensive care unit patients). About the disease course, critical COVID-19 patients showed four subsequent patterns (progression, absorption, enlargement, and further absorption) in our collected dataset, with remarkable concurrent HU patterns for GGO and consolidations.

**Conclusions:**

By segmenting the infection regions with a VB-Net and registering all the CT images and the segmentation results onto a template, spatial distribution patterns of infections can be computed automatically. The algorithm provides an effective tool to visualize and quantify the spatial patterns of lung infection diseases and their changes during the disease course. Our results demonstrate different patterns between COVID-19 and CAP, between severe and critical COVID-19, as well as four subsequent disease course patterns of the severe COVID-19 patients studied, with remarkable concurrent HU patterns for GGO and consolidations.

**Supplementary Information:**

The online version contains supplementary material available at 10.1186/s12880-021-00588-2.

## Background

CT imaging plays an important role in understanding of lung infections from COVID-19 and subsequent treatment assessment. In recent studies, Ai et al. [1] showed that chest CT findings were correlated with reverse transcription polymerase chain reaction (RT-PCR) testing outcomes. Caruso et al. [2] found that the specificity of chest CT was low (56%) but the sensitivity was high (97%) in diagnosing COVID-19. Similarly, a low rate of missing diagnosis of COVID-19 (3.9%, 2/51) is reported in Li and Xia [3], and CT could be used for screening COVID-19 patients in complement to RT-PCR tests [4].

The common CT features of COVID-19 include peripheral ground-glass opacities (GGO) and consolidations with multi-lobe and posterior involvement, bilateral distribution, and sub-segmental vessel enlargement [2]. Studies showed that multiple small patchy shadows and interstitial changes could appear in the outer lateral zone of the lungs in the early stage. Traction bronchiectasis often appears in the GGO area, and the formation of the subpleural band may cause structural distortion in some cases; as the disease progresses, multiple GGO and infiltration could appear in both lungs; in severe cases, pulmonary consolidation may occur, and pleural effusion is rare. Interlobular septa thickening and coincide of the intralobular line and GGO, called crazy paving sign, often appear with the disease progresses. Other imaging signs include bronchiectasis and pleural thickening, but pleural effusion, pericardial effusion, enlargement of lymph nodes, pulmonary cavity, CT Halo sign and pneumothorax are rare. These imaging features have been studied to distinguish the severity of the disease [5, 6] or to assess different stages of the disease during its time course [7–9]. For example, Bernheim et al. [7] studied 121 symptomatic patients and found that bilateral lung involvement occurred as the disease progressed. For patients without severe respiratory distress during the disease course, abnormalities on chest CT reach to peak severity approximately 10 days after initial onset of the symptoms and gradually recover thereafter [9]. However, these studies are mostly based on *qualitative* assessment of the images, and also limited by the relatively small number of recruited patients. As the purpose of this paper is to analyzing the spatial and temporal distribution patterns of infection regions by automatically segmenting and aligning images, we demonstrated the most important features such as GGO, consolidation and HU distributions and their subsequent changes of the population studied.

Constructing the distribution maps from a large number of imaging data is capable of depicting anatomical structures or functional uptake and is a promising way to demonstrate population-level findings. One may observe the physiologic changes of disease by comparing the spatial distribution pattern of the diseased cases with that of the normal cases. Meanwhile, a temporal sequence of the distribution maps across the time course of the disease are capable of tracking the progression and recovery of the disease. To our best knowledge, there is no attempt till now to construct such a group imaging pattern for COVID-19 to describe the infection in lungs and to delineate the progression of COVID-19 in a quantitative way.

To better understand the spatial pattern of COVID-19 infections in lungs at the population level as well as its evolution in the disease course, this study aims to construct the CT image distributions from a large COVID-19 patient cohort and models the spatial–temporal distribution of the infection regions using an automatic segmentation and registration pipeline. Specifically, we perform automatic contouring of the infection regions in the lungs and register all the chest CT images onto a common image space. Then, the spatial distribution of the infection regions can be computed in this common space. The distributions corresponding to different lesion sets or groups of different severity of COVID-19 at multiple time points alongside the time course of the disease, and against that of CAP, are compared quantitatively.

For this purpose, hundreds and thousands of the CT images need to be segmented, which is a tedious and time-consuming work. Thus, we present a neural network called VB-Net to segment the infection regions. To train the model, a human-involved-model-iterations (HIMI) strategy is adopted to iteratively segment and manually correct the infection regions. Basically, two radiologists annotate a group of images initially and then the neural network is trained using these images. Then, more training images are added by first applying the model and then correcting the segmentation manually. In this way, the training samples can be gradually annotated to update the model training procedure. Totally, we used 249 CT scans for training and 300 separate images for validating the segmentation model. The validation dataset was generated without applying the HIMI strategy. Then, the VB-Net was used to process the rest of the data of this study. After image registration, the spatial distribution maps and their time-course changes are computed for further analysis.

In this paper, we demonstrate that by automatically segmenting and registering the images and infection regions and constructing the spatial distribution of infection in a template CT image, patterns of infections can be visualized and quantified for comparison of different groups. The comparison results and patterns changes during the disease course provide better understanding of COVID-19 based on our dataset.

## Methods

### Data collection

249 CT scans of 249 COVID-19 patients were collected from hospitals outside Shanghai, China for training the segmentation network, and 300 CT images were collected from the Public Health Center in Shanghai for validation (for those patients not admitted to ICU). All the COVID-19 patients were confirmed with a laboratory examination through real-time PCR (RT-PCR) detection by the local Center for Disease Control (CDC), and rechecked by national CDC. The scanners used include UCT780 from UIH, Optima CT250, LightSpeed 16 from GE, Aquilion ONE from Toshiba, SMMATOM Force From Siemens, and Scenaria 64CT from Hitachi. The major parameters of CT protocols are: 120 kV; automatic tube current (180–400 mA); iterative reconstruction; rotation time around 0.35 s; collimation of 0.625 mm; pitch of 1.5; axial plane matrix of 512 × 512; and breath hold at full inspiration. The reconstruction kernel used is set as lung window per definition of manufactures. During reading the lung window (with window width 1200 HU and window level 600 HU) was used. All the scans are normal CT without contrast enhancement, and all the CT scans are captured in thin-section (< 2 mm). We selected the patients with a positive new coronavirus nucleic acid and confirmed by the CDC of China. All the selected patients are with age above 18 and the chest CT show infection. Only the scans reconstructed by lung window are used. We also excluded the duplicated scans of the same patient obtained at the same day. Two radiologists independently annotated the validating data and involved in the HIMI training procedure of the segmentation model.

To construct the disease distribution patterns of COVID-19 and CAP, we used 2954 images from 2760 COVID-19 patients confirmed with positive RT-PCR by following the protocols for the Diagnosis and Treatment of COVID-19 (Trial revised version 5), and 1343 images from 1089 CAP patients. The spatial distributions of ground glass opacification (GGO) and consolidation were also constructed and compared.

For progression and recovery course study, additional 457 longitudinal CT images of 83 critically ill patients with intensive care unit (ICU) events and 1236 images from 223 severe patients without ICU care were used for time course analysis, including lesion distributions of the onset CTs when the diagnosis of COVID-19 was confirmed. All the cases used for progression study are from Shanghai Public Health Clinic Center, which have at least one follow-up scan. Because of low modality rate in Shanghai, we excluded a few death cases. We classified the data into severe patients (inpatients without ICU care) and critical patients (with ICU care) according to the protocols for the Diagnosis and Treatment of COVID-19 (Trial revised version 5), which was issued by the National Health Commission of China on Feb. 4th, 2020. Specifically, severe cases are those with obvious lesion progression (> 50% increase within 24–48 h) and (1) respiratory distress (≧ 30 breaths/ min); (2) oxygen saturation ≤ 93% at rest; (3) arterial partial pressure of oxygen (PaO_2_)/fraction of inspired oxygen (FiO_2_)≦300 mmHg (l mmHg = 0.133 kPa). Critical cases are the patients who require ICU care. As the longitudinal data are scanned between Jan. 20th, 2020 and Mar. 6th, 2020, we double checked the clinical criteria of all these patients underwent ICU treatment to make sure the grouping are correct.

Ethical approval was obtained from the ethics committee of the Shanghai Public Health Clinical Center (IRB No. 2020-E015-01, March 26, 2020). Written informed consent was waived because only anonymized imaging data and ICU dates were used. The study did not alter any diagnostic and treatment decisions of the patients included. All the methods were performed in accordance with the relevant guidelines and regulations.

### Automatic infection segmentation using VB-Net

Automatic segmentation of infection regions could be affected from the low contrast of the infection regions manifested as GGO in CT images and large variation of both shape and position across different patients. Our group developed a DL-based network called VB-Net, for lung CT image segmentation [10]. The VB-Net is a modified 3D convolutional neural network that combines V-Net with the bottle-neck structure, consisting of two paths. The first path is a contracting path including down-sampling and convolution operations to extract global image features. The second path is an expansive path including up-sampling and convolution operations to integrate fine-grained image features.

In the contracting path, the number of channels after the first convolution layer is 16. Each down-sampling layer doubles the number of channels doubles and halves the size of feature maps. On the other hand, the number of channels decreases by half, and the size doubles after each up-sampling layer. Skip connections are used at each resolution by employing a concatenating operation.

At each level, three convolution layers are used. Herein, we replace the 5 × 5 × 5 convolution operation in the V-Net by a bottleneck structure. Specifically, the first convolutional layer reduces the channels of feature maps by applying 1 × 1 × 1 convolution kernel. The second convolutional layer performs spatial convolution with 3 × 3 × 3 kernel. The last convolutional layer increases the channels of feature maps by applying 1 × 1 × 1 convolution kernel. Compared with V-Net, the speed of VB-Net is much faster and with significantly smaller GPU memory.

### Computing spatial distributions of infections

**Fig. 1:**
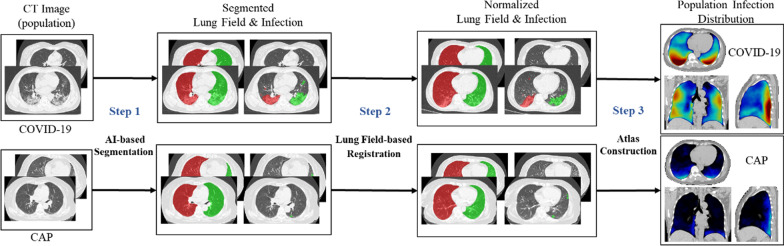
The pipeline to construct respective distribution maps for the COVID-19 and CAP image populations. Step 1 is an AI-based lung field and infection region segmentation network VB-Net; step 2 registers all the images and infection regions onto a common template image; and step 3 computes respective spatial distributions of infection regions

As shown in Fig. 1, three steps were adopted to build the distribution maps. First, the VB-Net was used to segment the lung fields and infection regions. The segmentation network achieved an average Dice coefficient of 91.6% between automated and manual segmentations of the infection regions for COVID-19, and generated satisfactory segmentations for the rest of the images studied in the paper. Second, we registered all the CT images based on segmented lung field masks to a template through affine and deformable registration [11, 12]. The template is a normal CT image whose lung volume is close to the median of the population data studied. By warping the segmented lung field masks (left and right) of each subject to the template with nearest neighborhood interpolation, the infection regions of each subject are obtained in the template space. Finally, the distribution maps were constructed to encode spatial distribution of the normalized infection regions.

### Construction of distribution maps

The distribution map, which reflects the distribution map of the infection regions, was constructed by first accumulating the binary infection masks in the template image space and then dividing the result by the number of images. Thus, the value at each voxel location denoted the probability of infection, which was the ratio of the infected lungs at that voxel to the total number of the lungs under consideration.

Specifically, suppose the template image is $T$, the infection region of subject $i$ is ${S}_{i}$, $i=1,\dots ,N$, and the deformation field is ${F}_{i}$, the warped segmentation is calculated by ${S}_{i}^{^{\prime}}={F}_{i}\circ {S}_{i}$ with nearest neighborhood interpolation. The distribution map of the infection regions is calculated as $m=1/N{\sum }_{i}{S}_{i}^{^{\prime}}$. Using the same computing method, we constructed multiple distribution maps corresponding to different segmentation regions or groups as follows:

- The COVID-19 map (calculated using 2954 COVID-19 images) and the CAP map (calculated from 1343 CAP images).
  - The COVID-19-GGO, COVID-19-Consolidation, CAP-GGO, and CAP-Consolidation maps, obtained by separating the warped infection regions into GGO and consolidation components, i.e., with an HU threshold − 300 (GGO < − 300, and consolidation $\ge -$ 300), on the corresponding normalized CT images.
- Onset scan of severe and critical COVID-19 patients.
- Longitudinal distribution maps for critical COVID-19 patients (457 images of 83 patients) were also computed by first aligning the scanning dates to the dates of ICU admission and then grouping the images into nine time-course stages.

### Histograms of infection regions

To illustrate the intensity distribution of infections, we calculated the histograms of HUs in infection regions for each subject, and then average them across all subjects. Histograms were computed from the original CT images rather than the warped CT images. The reason is that deformable registration cannot preserve voxel volumes and often presents interpolation and partial volume effects for HU values. Note that only HU values within the range of − 1000 ~ 150 were calculated, and 128 bins were used for calculating the histograms. The population-wise histograms were calculated from the image intensities within all the respective infection masks.

### Statistical analysis

Voxel-wise ${\mathcal{X}}^{2}$ test was used to compare the difference of infection occurring frequencies between two maps. Specifically, at each voxel location, the *p* value was calculated by comparing the frequencies of the voxels infected in two respective distribution maps.

As HU histograms were calculated for infection regions of different image groups, the Kolmogorov–Smirnov (K–S) test [13] was used to compare whether the two histogram distributions are significantly different. The null hypothesis is that two independent HU histograms are drawn from the same continuous distribution. If the *p* value is significant, then we cannot reject the hypothesis that the distributions of the two groups are the same.

All statistical analyses were performed using Python toolbox SciPy. The significant level was set to 0.05. Notably, given the potential for type I error, the false discovery rate (FDR) could have been used to adjust multiple comparisons. Thus, the findings by using simple *p* value thresholding should be interpreted as exploratory and descriptive.

## Results

### Performance of infection region segmentation

By segmenting the 300 validating CT images, the average Dice is 91.6% ± 10.0%, and for the same dataset the inter-rater variability analysis between two radiologists indicates that the average Dice coefficient is 96.1% ± 3.5%. By comparing with a V-Net segmentation (with average Dice 87.3% ± 10.1%) the segmentation performance of VB-Net has been improved significantly (*p* < 0.001). For annotating the training images, we performed three round of training and correcting of the training samples, and the average annotating time was reduced to around 4.7 min per case from 211 min per case, showing a significant time reduction when drawing the masks for infection regions. Notice that to have a fair comparison, the annotation of the validating dataset was performed by two independent radiologists and did not refer to any automatic segmentation results.

### Comparison between distribution maps of COVID-19 and CAP

**Fig. 2:**
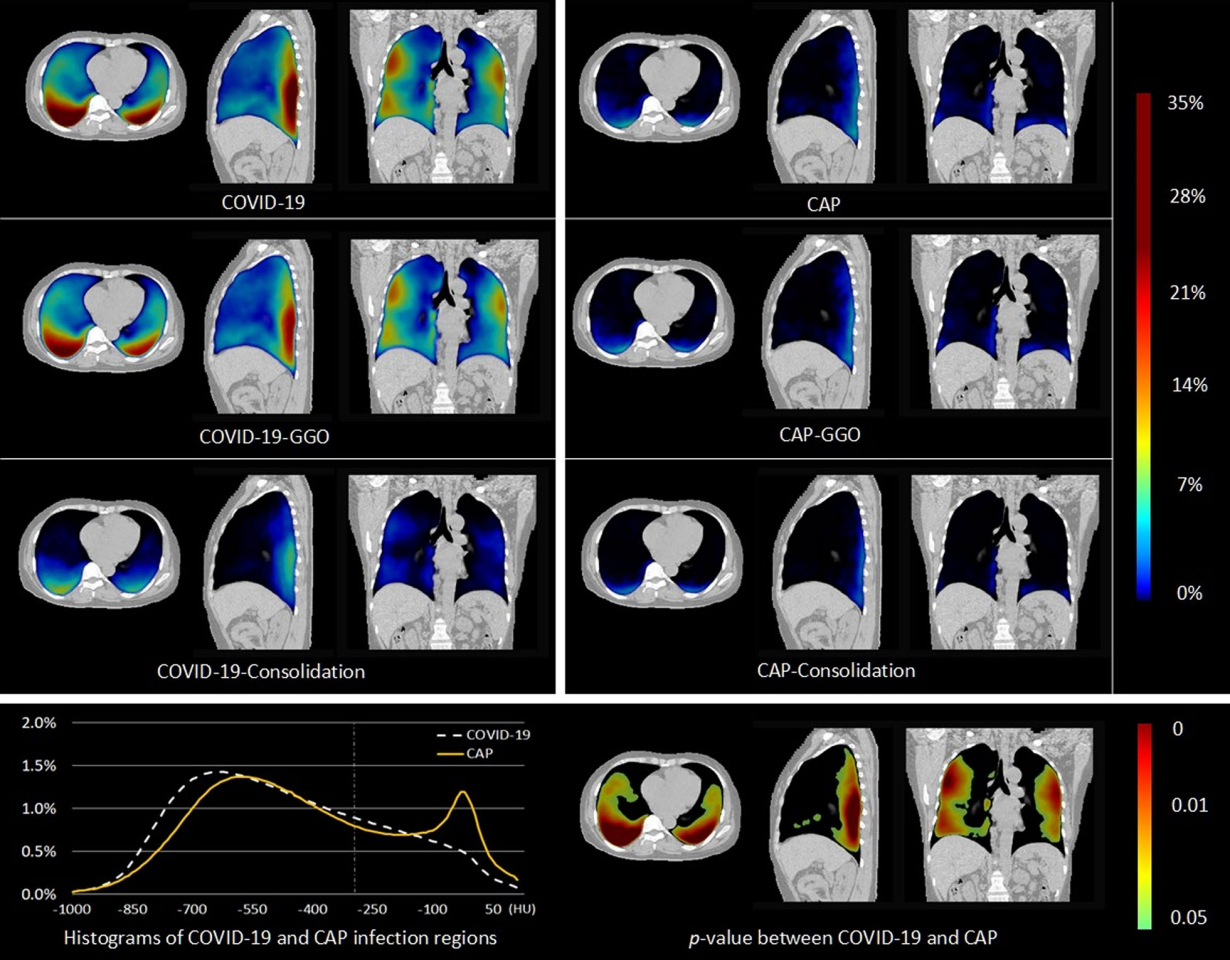
The distribution maps for COVID-19 (first row, left) and CAP (first row, right), GGO and consolidation distribution probabilities (2nd and 3rd rows), HU distributions of COVID-19 and CAP infections (bottom row, left). These maps show the spatial distributions of the infection occurring frequency. The *p* value map (bottom row, right) highlights the locations with significant difference between COVID-19 and CAP distributions

The distribution maps for the spatial distribution of infections of (1) 2954 CT images of 2760 COVID-19 patients (first row, left of Fig. 2) and (2) 1343 images of 1089 CAP patients (first row, right of Fig. 2) were calculated. The COVID-19 infections have the occurring frequency up to 35.1%, and mostly distributed on peripheral, posterior, and middle-lower pulmonary lobes. On the contrary, the highest infection frequency count was much lower (8.9%) as reflected by the CAP map. In general, the CAP induced infections were smaller in overall size or number. Also, the CAP infections tended to be peripheral and close to the diaphragm.

Figure 2 also shows GGO (second row) and consolidation (third row) infection distributions of COVID-19 and CAP. For COVID-19, GGO infections held a large percentage of infected regions and spread more widely than consolidation infections. In COVID-19, the maximum probabilities of GGO and consolidation components were 26.1% and 10.7%, respectively. In CAP, these two types of infections were relatively mild, with the maximum probability of 7.9% in GGO and 5.1% in consolidation.

The *p* value map between infection frequencies of COVID-19 and CAP was calculated using ${\mathcal{X}}^{2}$ test and shown on the bottom (right) of Fig. 2. This *p* value map highlights the locations where two maps and their underlining patient cohorts have significant difference. The locations with *p* < 0.05 accounted for 48.0% of the total lung field volume, indicating that the locations and sizes of COVID-19 infections were prevalently and significantly different from CAP in the lung. We further computed the ratios of the volumes of significantly different voxels in the right and left lungs, as well as five lobes over the entire lung volume, and reported them in Table 1. The most different region is the right lung, especially the right-lower lobe (bold font in Table 1). The left-upper lobe is another affected region with *p* value < 0.05, with the volume of voxels being 12.8% of the entire lung. We also compared the percentage of the region with *p* value < 0.05 with respect to the left/right lungs or lobes that the infection locates.

**Table 1:** Ratio between the volume of statistically different regions (with *p* < 0.05) and the total volume of lung/lung lobe, and left/right lung and lobes, compared between COVID-19 and CAP in Fig. 2 and between severe and critical COVID-19 in Fig. 2, respectively

Region with *p* < 0.05 between two groups (A vs. B)	Volume percentage in lungs			Volume percentage in lobes				
	Lung (%)	Lung-R (%)	Lung-L (%)	Upper-R (%)	Middle-R (%)	Lower-R (%)	Upper-L (%)	Lower-L (%)
COVID-19 versus CAP (divided by whole lung volume)	48.0	28.3	19.7	10.2	6.1	12.0	12.8	6.9
Severe versus Critical (divided by whole lung volume)	0.1	0.1	0	0	0	0.1	0	0
COVID-19 versus CAP (divided by respective volumes)	48.0	50.6	44.6	42.8	42.0	71.0	41.4	56.0
Severe versus Critical (divided by respective volumes)	0.1	0.2	0	0	0	0.7	0	0

To further analyze the HU distributions, we calculated the histograms of HU values within the infection regions of all the samples of the two groups (bottom left of Fig. 2). For statistical analysis, K-S statistics was employed to compare the histograms of two groups. It turns out that, by splitting the histograms using HU threshold − 300 (as shown by the vertical dotted-dashed line), the *p* value of the GGO part (HU < − 300) was 0.42, and the *p* value of the consolidation part (HU $\ge$− 300) between COVID-19 and CAP was 0.0005. Thus, comparing with CAP, COVID-19 had significantly less consolidation components.

### Comparison between onset stages of severe and critical COVID-19 cases

The severe COVID-19 cases were those undergoing inpatient treatments without going to ICU, and the critical COVID-19 cases were those admitted to ICUs (as described in the data collection subsection). All selected cases finally recovered after hospitalization. The above patient cohort allows us to observe the difference of infection patterns between severe cases and critical cases. Because it is highly interesting to see whether these patients’ CT images are different from the initial stage of the illness, we hereby chose to use the first images captured when the patients were diagnosed and prior to ICU admission if any, for comparison.

**Fig. 3:**
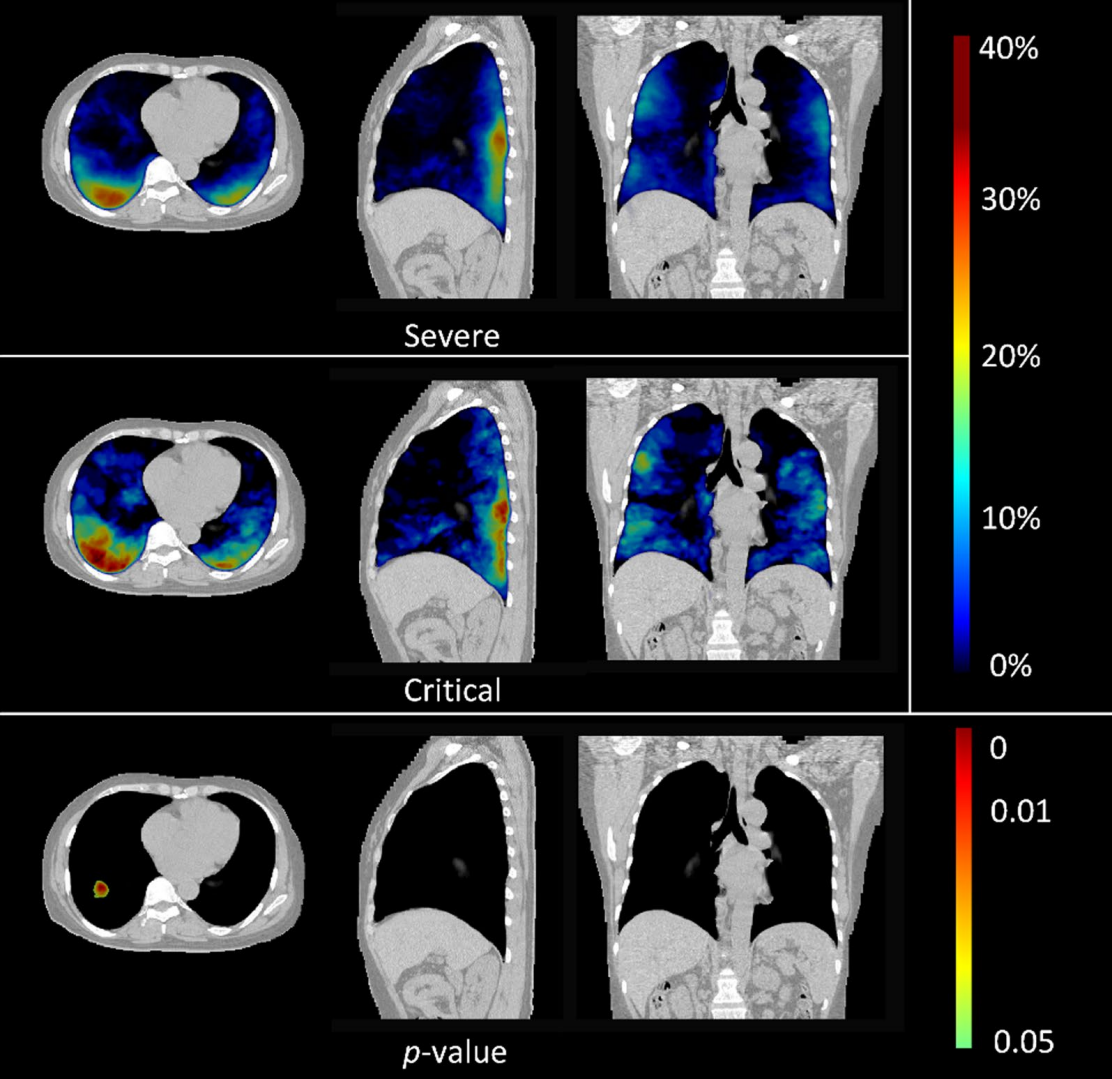
Comparison between onset images of severe and critical COVID-19 cases

Figure 3 shows the lesion distributions for severe (top) and critical (middle) cases at their first scan, and the *p* value maps calculated using K–S test are shown on the bottom. Each group had 45 images from different subjects. The two distribution maps were quite similar, and the region with significant difference was also small (i.e., counting for 0.1% of the lung volume). This might partially explain why it is difficult to predict severity with onset images, although it is invaluable for exploring such a prediction. Table 1 gives the ratios between the volume of significantly different region to the total lung volume. The right lung, especially the right-lower lobe, had relative difference (0.1%).

### Time course of critical COVID-19 patients

**Fig. 4:**
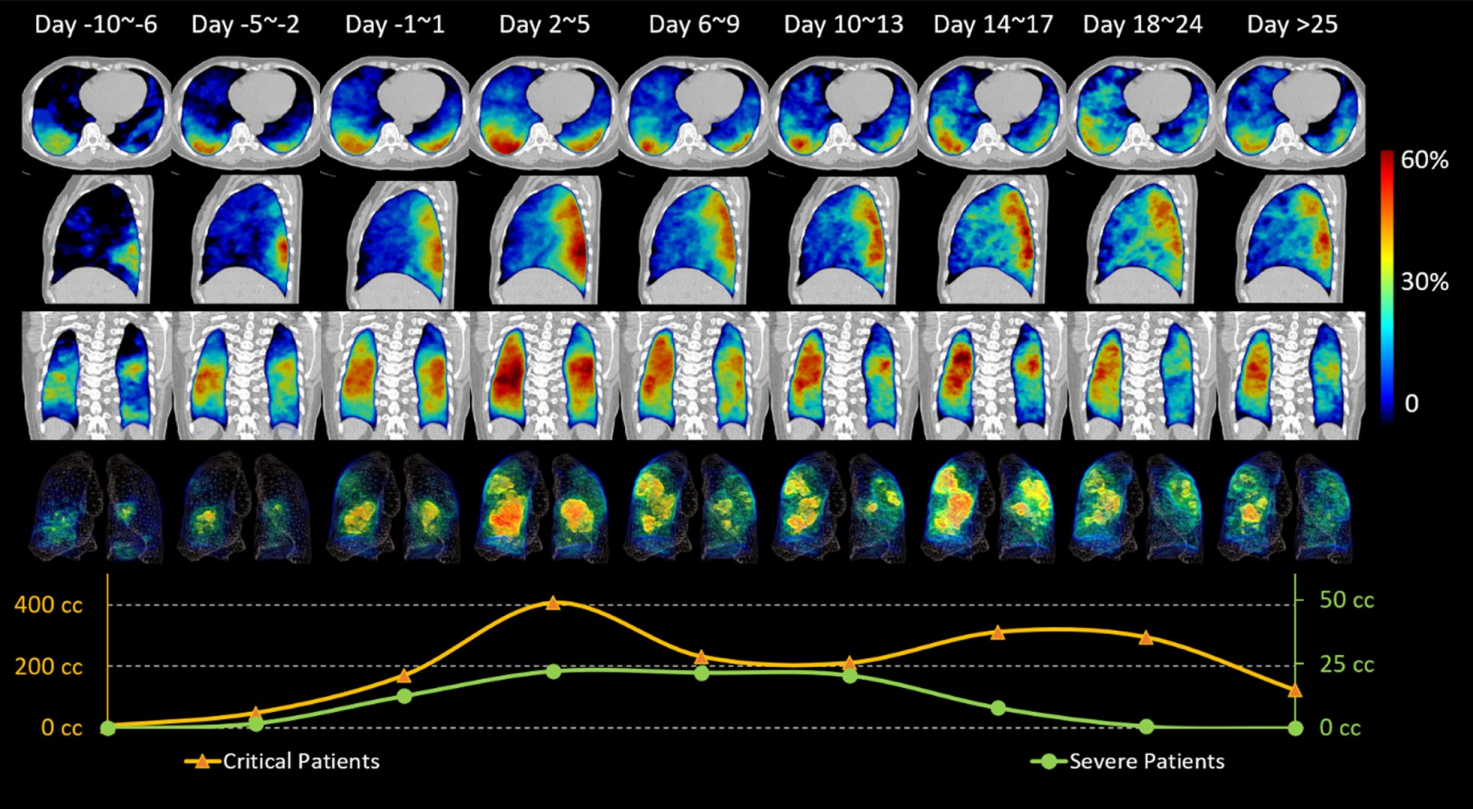
Time course of critical COVID-19 patients. The first three rows show the axial, sagittal and coronal views of infection distributions in 9 stages/groups, along with their 3D renderings shown in the fourth row. The last chart shows the total volume of infection regions (with infection probability greater than 35%) in each of 9 stages/groups of critical and moderate patients

To assess the images of critical patients during the disease course, the spatial–temporal infection distributions were calculated and shown in Fig. 4. For each patient, all images were temporally sorted based on Day 0, which was defined as the date admitting the patient into ICU. Nine groups along the time course were identified for a total of 457 images (83 patients): Day − 10 ~ − 6, n = 33; Day − 5 ~ − 2, n = 54; Day − 1 ~ 1, n = 79; Day 2 ~ 5, n = 82; Day 6 ~ 9, n = 59; Day 10 ~ 13, n = 39; Day 14 ~ 17, n = 35; Day 18 ~ 24, n = 34; and Day > 25, n = 42. Figure 4 shows that the infections rapidly grew and reached the peak at 2 ~ 5 days after being admitted to ICU (Day 0). In our cohort of patients we have observed this evolution: the infections started to absorb in Day 6 ~ 13, yet enlarged again in Day 14 ~ 17. After 18 days, the infections were slowly absorbed. The above findings further confirm that COVID-19 progresses rapidly and recovers slowly for severe cases.

Similarly, we also grouped the time course images of severe patients into nine stages and computed their infection distributions accordingly. The volume curves of the regions with high infection probability in both critical and severe patient groups are calculated using a threshold (35%) and plotted on the bottom of Fig. 4. It can be clearly seen that the distribution maps have demonstrated the progression (Day − 10 ~ 5), absorption (Day 6 ~ 13), enlargement (Day 14 ~ 17), and further absorption (Day > 17) stages for critical patients, while the curve of severe patients shows gradual increase in first 10 days and then decrease thereafter. And meanwhile, the volume of high infection probability in severe patients is smaller (peak volume less than 25 cc) than critical patients (up to 400 cc).

**Fig. 5:**
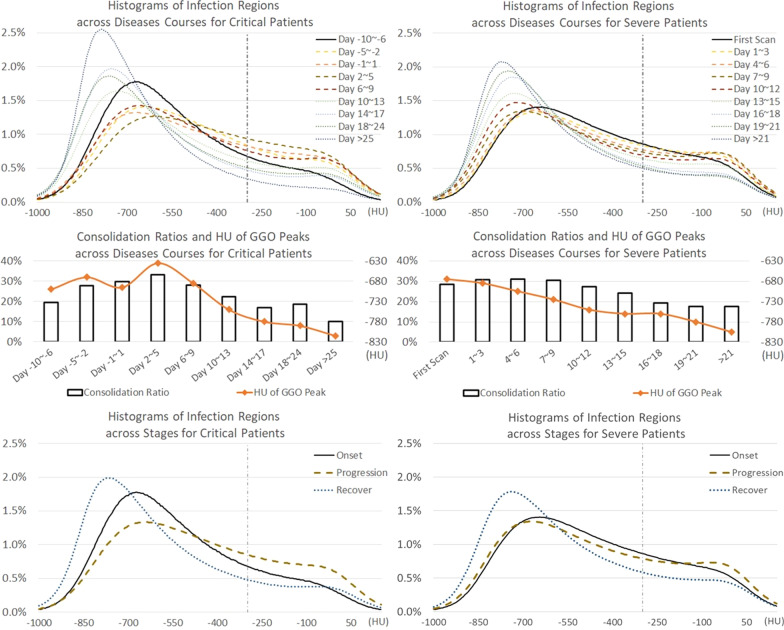
Comparison of HU distributions, consolidation ratios, and GGO peak HU values of critical (left) and severe (right) patient groups. Top: the HU distributions of nine time-course stages; middle: consolidation ratios and the HU of GGO peaks; bottom: HU distribution of the onset, progression, and recovery stages

Figure 5 (top) shows the HU distributions of the nine time-course groups of severe (right) and critical (left) patients. We used black solid curve to represent the earliest HU distribution, and four dashed curves and four dotted curves of critical patients (left, top) shows the progression and recovery stages, respectively. It can be seen that GGO and consolidation changes are clearly associated with the disease time course. Similarly, the top-right part of Fig. 5 is the HU distributions of severe patients. The black solid curve is the average histogram at the first time-point. The consolidation in severe patients also tends to increase initially and then gradually decrease. The overall variation of severe patient is smaller compared to critical patients.

Figure 5 (middle) plots both the ratios of consolidation lesions (i.e., the areas under the curves for HU greater than − 300) and the HU values of the GGO peaks. The time course of the ratios of consolidation in critical patients (the middle-left plot) is consistent with the aforementioned progression, absorption, enlargement, and further absorption stages; and the HU values of GGO peaks consistently shift after reaching the peak (severe) stage. The ratios of consolidation in severe patients (the middle-right plot) first increase and then gradually decrease, and the GGO peak HU values are smaller than those of critical patients and show a trend of overall gradual decrease across with time. The videos showing time course of COVID-19 (severe stage) are in the Additional files 1, 2 and 3.

After grouping the critical cases into the progression stage (dashed curves in Fig. 5) and the recovery stage (dotted curves in the bottom left of Fig. 5) and calculating their average histograms, the *p* values of K–S tests among the three distributions were 0.0004 between progression and recovery stages, 0.008 between onset and progression stages, and 0.36 between onset and recovery stages, respectively. After splitting the GGO and consolidation parts, the p-values of GGO lesions were 0.0005 between progression and recovery stages, 0.0003 between onset and progression stages, and 0.26 between onset and recovery stages, respectively. For the consolidation parts, all the K–S tests showed significant difference (*p* value < 0.05) for all combinations. These results supported the possibility of identifying respective image features of these stages and correlating them with clinical measures. Similar grouping of severe patients is shown in the bottom right picture of Fig. 5. We summarized major findings obtained from the experiments of this research in Table 2.

**Table 2:** A summary of major information obtained from this research

Studies	Major findings from the experimental results in our cohort of patients
Comparison of infection distributions of COVID-19 and CAP	The locations and sizes of COVID-19 infections are prevalently and significantly different from CAP in the lung COVID-19 mostly distributed on peripheral, posterior, and middle-lower pulmonary lobes CAP infections are smaller in size or number, and tend to be peripheral and close to the diaphragm
Comparison of GGO and consolidation distributions between COVID-19 and CAP	In COVID-19, GGO infections hold a large percentage of infected regions and spread more widely than consolidation infections. The maximum probability of GGO and consolidation are 26.1% and 10.7%, respectively In CAP, these two types of infections were relatively mild, with the maximum probability of 7.9% in GGO and 5.1% in consolidation
Comparison of onset stages in severe and critical COVID-19 cases	The two distribution maps are similar The region with significant difference was small (counting for 0.1% of the lung volume) The right lung, especially the right-lower lobe, has relative difference in the two distributions
Time course of critical COVID-19 cases	GGO and consolidation changes are clearly associated with the disease time course The critical COVID-19 patients showed 4 subsequent patterns: progression, absorption, enlargement, and further absorption, with remarkable concurrent HU patterns for GGO and consolidations

## Discussion

CT imaging is an important supplemental tool to assess the severity of COVID-19, but radiologists lack computational means to quantify the images. This paper presents simple yet effective pipeline to first segment the infection regions, register the images onto a common template space, and then compute the quantitative distributions of the infection regions of different types of infection regions. The distributions across the disease time course can also be visualized and quantified based on the same method. The patterns of infection and their changes could help better understand the differences between corona virus infections and others and also illustrate the recovering time course of COVID-19.

The methodology established in this work could also be applied to compare between other groups, i.e., subjects undergoing different treatments and subjects not recovering from the disease, to better understand the underlying difference in terms of imaging features. Similar methodology is also applicable to other pathologies.

Beside population-level spatial patterns of the disease, HU distributions were examined and compared longitudinally. Nevertheless, although group comparison provides the knowledge of population and their differences, it does not directly indicate automatic identification of singular samples using descriptors such as HU distribution, but pin-points the regions of difference for possible classification. In the future works, features derived from group comparison could be used for designing a classifier.

Using image segmentation and registration techniques, lung fields and infection regions of different subjects were registered onto a common template space to analyze the spatial distribution of infections for a large number of subjects. Only lung field masks were used to drive the registration in this work. The lungs and infections were spatially normalized based on the resulting deformation fields. Thus, the deformations within the lungs are smooth, estimated mainly by lung surfaces. This may render alignment discrepancies of internal structures. However, given a large number of imaging data, the group maps still reflect how infections distribute within the lung field. In the future, deformable registration that considers the alignment of internal structures such as major normal airways and vessels is needed to more accurately align infections across subjects. Infection regions could be exempted when calculating such a registration because of their variability.

Power analysis showed that, in order to obtain 90% statistical power from K–S test at a significant level of 0.05, forty-five samples were required. In this study, we grouped the time course of 457 images of 83 subjects into 9 groups, with some groups having large sample size. As a result, the p-values across different time course stages were not computed. On the other hand, the numbers of samples for comparisons between COVID-19 and CAP and also their HU histograms provide sufficient statistical power (> 90%) for voxel-wise ${\mathcal{X}}^{2}$ test and K–S test, respectively. For example, for a middle effect size of 0.3, we need 117 samples for two category ${\mathcal{X}}^{2}$ test at significance level 0.05. In this paper, we have 2954 COVID-19 and 1343 CAP image samples, and the statistical power is close to 1. These results did stably show regions with significantly different spatial distribution patterns of infections between COVID-19 and CAP, and between severe and critical COVID-19, as well as consistent HU shifting in GGO and the amount shifting in consolidation during the disease time courses of critical COVID-19 patients.

The proposed lesion maps can be considered as the normalized counts of all the warped lesion masks, which could be affected by multiple factors, including the number and size of masks, number of samples, as well as their shapes. Therefore, bigger probability at a voxel location could indicate that the voxel location occurs more often inside the infection regions, and also a continuous region with higher probability in the distribution maps could be contributed either by bigger lesions or spread smaller lesions covering that region.

As mentioned in Background, CT imaging patterns of COVID-19 patients include vascular and bronchial pathology beside infections. In this study, as our objective was to analyzing the spatial and temporal distribution patterns of infection regions by automatically segmenting and aligning images, we only compared the infection distributions between COVID-19 and CAP, between severe and critical in COVID-19 onset stages, as well as the time course of critical COVID-19 patients. We will analyze other imaging patterns in the future work.

## Conclusions

Population-based infection maps were constructed in this paper by employing a pipeline of automatic segmentation, registration and distribution computing, for different infection regions of the lung. Using statistical tests the distributions of COVID-19 and CAP were compared. Additional distribution maps were also constructed, corresponding to onset stages of severe and critical COVID-19, and for individual stages along the time course of critical COVID-19. Comparisons among these maps showed that the infection regions of COVID-19 were more widely spread in the lung than those of CAP, although both distributions are peripheral. The time course of critical COVID-19 demonstrated progression, absorption, enlargement, and absorption stages in CT images, showing rapid acceleration and slow recovery, with consistent shifting of GGO and consolidation, while the relative mild group demonstrate only gradual increase and degrease stages during the disease course. Severe and critical patients had similar population-level distribution of lesions at onsets yet with small areas of significant difference, rendering possible but difficult for severity prediction in early assessment of the disease.

## Supplementary Information

**Additional file 1.** Time course of COVID-19_1.

**Additional file 2.** Time course of COVID-19_2.

**Additional file 3.** Time course of COVID-19_3.

## Data Availability

The datasets used and analyzed during the current study are available from the corresponding author on reasonable request.

## Abbreviations

CAP: Community acquired pneumonia
COVID-19: Coronavirus disease 2019
CT: Computed tomography
FDR: False discovery rate
GGO: Ground glass opacification
HU: Hounsfield unit
ICU: Intensive care unit
IRB: Institutional Review Board
K–S test: Kolmogorov–Smirnov test
RT-PCR: Reverse transcription polymerase chain reaction
K–S test: Kolmogorov–Smirnov test
